# Constructing Cu_7_S_4_@SiO_2_/DOX Multifunctional Nanoplatforms for Synergistic Photothermal–Chemotherapy on Melanoma Tumors

**DOI:** 10.3389/fbioe.2020.579439

**Published:** 2020-09-15

**Authors:** Leilei Zhang, Hui Pan, Yongyun Li, Fang Li, Xiaolin Huang

**Affiliations:** ^1^Department of Ophthalmology, Ninth People’s Hospital, Shanghai Jiao Tong University School of Medicine, Shanghai, China; ^2^Shanghai Key Laboratory of Orbital Diseases and Ocular Oncology, Shanghai, China

**Keywords:** Cu_2–x_S nanocrystals, mesoporous SiO_2_, photothermal therapy, chemotherapy, melanoma

## Abstract

The integration of photothermal therapy and chemotherapy has been recognized to be an efficient strategy through the instant thermally ablation and long-term chemical inhibition, thus achieving high therapeutical effect. In the present work, we designed and prepared Cu_7_S_4_@SiO_2_/DOX nanocomposites and used them as efficient nanoplatforms for synergistic photothermal-chemo therapy on melanoma tumors. The Cu_7_S_4_@SiO_2_/DOX was constructed by firstly synthesizing Cu_7_S_4_ nanocrystals, then *in situ* growing SiO_2_ shell on the surface of Cu_7_S_4_ nanocrystals, and finally loading DOX within SiO_2_ shell. The Cu_7_S_4_@SiO_2_/DOX was composed of Cu_7_S_4_ core as the photothermal transducer, SiO_2_ shell as DOX carrier and DOX as the model of anticancer drug. Once exposed to a 1064 nm laser, the Cu_7_S_4_@SiO_2_/DOX could simultaneous generate heat for photothermal therapy and accelerate the DOX release. When the Cu_7_S_4_@SiO_2_/DOX was injected into the center of tumor, the tumor exhibit rapid temperature elevation once exposed to the NIR laser and the tumor growth is significantly inhibited through the synergistic photothermal-chemo therapy, in comparison to the limited therapeutical effect of photothermal therapy or chemotherapy alone. Therefore, the Cu_7_S_4_@SiO_2_/DOX with photothermal-chemo function can be used as excellent nanoplatforms for treating solid tumor with high theoretical effect.

## Introduction

The near infrared (NIR) light-driven cancer treatments have caught a numerous attention for years due to the NIR light with higher tissue-penetration depth than visible light and the better safety than ultraviolet light (Cheng et al., 2014; Vankayala and Hwang, 2018). Among these NIR-induced therapy modalities, the photothermal therapy is an emerging one that utilizes NIR absorbents as energy transducers to convert NIR laser energy into heat (>42°C), so that thermally ablate cancer cells (Zou et al., 2016; Yu et al., 2018b). For the development of photothermal therapy, the photothermal nanoagents are the key point and they should be capable of strong and broad NIR photoabsorption and high photothermal conversion efficiency. Apart from the noble metal nanostructures (Dreaden et al., 2011; Huang et al., 2011) and the organic-based nanoparticles (Liu et al., 2013; Zha et al., 2013), the recent progress of photothermal nanoagents focus on semiconductor nanomaterials because of their abundant types, tunable composites, photostability as well as efficient photothermal effect (Huang et al., 2017). The semiconductor-based photothermal nanomaterials mainly include the metal oxides such as the doped TiO_2–x_ nanocrystals (Ren et al., 2015; Ou et al., 2016; Yu et al., 2017) and oxygen-deficient WO_3–x_ nanocrystals (Xu et al., 2015; Wen et al., 2016), and the metal sulfides including Cu_2–x_S nanostructures (Tian et al., 2011a,b) and Bi_2_S_3_ nanodots (Li et al., 2016). For example, Cu_7.2_S_4_ nanoparticles were prepared by the thermolysis of Cu(DEDTC)_2_ precursors and they exhibited strong NIR absorption, photostability and photothermal conversion efficiency up to 56.7%, which were used as a photothermal nanoagent for the photothermal ablation of cancer cells (Li et al., 2014). However, the therapeutical effect of photothermal therapy is executed only under the illumination of laser and it would disappear instantly when laser is switched off. Thus, to achieve the long-term therapeutical effect for photothermal nanoagents, it is quite necessary to combine with other therapeutical modalities.

The integration of photothermal therapy and chemotherapy has been recognized to be an efficient strategy through the instant thermally ablation and long-term chemical inhibition (You et al., 2012; Yu et al., 2018a; Zhang et al., 2020). Importantly, the photothermal effect can accelerate the drug releasing rate and induce the chemo-sensitization effect, thus achieving the synergistic effect with higher therapeutical results than photothermal therapy or chemotherapy alone. Up to data, there are a number of nanoagents with the photothermal effect and drug loading capacity, which can be allocated into two types. The first type consists the photothermal nanomaterials with a large volume of inner cavity or high specific surface area, such as CuS hollow nanospheres (Wang et al., 2018), two-dimensional MoS_2_ nanosheets (Liu et al., 2014) and metal-organic frameworks (Zhang et al., 2018). For instance, CuS hollow nanospheres with 87.7% of high doxorubicin (DOX) content were prepared while the DOX release rate at pH 7.4 was only 4.6% and at pH 5.0 was 10.3% within 10 h (Wang et al., 2018). The second type is the combination of photothermal nanoagent and drug carrier, which includes Cu_9_S_5_@mSiO_2_-PEG core-shell structures (Song et al., 2013), Au@copolymer-liposome nanostructures (Zheng et al., 2016) and CuS@gel nanocomposites (Meng et al., 2016). For example, a thermosensitive MEO_2_MA@MEO_2_MA-co-OEGMA nanogels were firstly prepared and then CuS nanoagents were *in situ* deposited within nanogels, whereas the DOX loading content was less than 10%, (Meng et al., 2016). Therefore, it is of great importance to design and synthesis of photothermal-chemo nanoagents with the high photothermal conversion efficiency and high drug loading capacity.

In order to integrate synergistic photothermal-chemo functions, we prepared a Cu_7_S_4_@SiO_2_/DOX. The Cu_7_S_4_@SiO_2_/DOX was constructed by firstly synthesizing Cu_7_S_4_ nanocrystals, then *in situ* growing SiO_2_ shell on the surface of Cu_7_S_4_ nanocrystals, and finally loading DOX within SiO_2_ shell. The Cu_7_S_4_@SiO_2_ nanoplatforms exhibited the strong and broad NIR absorption and could rapidly convert 1064 nm laser energy into heat with the efficiency of 48.2%, and they also demonstrated large specific surface area and pores with high DOX loading content of 59.8%. Importantly, After the irradiation cycles, 90.1% of DOX was released from Cu_7_S_4_@SiO_2_/DOX with the help of 1064 nm NIR laser at pH 5.4 in comparison to 61.5% of the released DOX without irradiation, indicating photothermal effect accelerated the DOX release. More importantly, when Cu_7_S_4_@SiO_2_/DOX was intratumorally injected into tumor-bearing mice, the tumor growth was heavily inhibited through the synergistic photothermal-chemo therapy compared with the limited therapeutical effect of photothermal therapy or chemotherapy alone. Therefore, the Cu_7_S_4_@SiO_2_/DOX with high photothermal conversion efficiency and drug loading capacity can be used for treating solid tumor with high therapeutical effect.

## Materials and Methods

### Materials

Sodium diethyldithiocarbamate (SDEDTC), CuCl_2_⋅2H_2_O (AR), oleic acid (AR), oleylamine (80–90%), cetyltrimethylammonium bromide (CTAB), sodium hydroxide (NaOH, AR), doxorubicin hydrochloride (DOX) and tetraethylorthosilicate (TEOS, GR) were brought from Sigma Aldrich.

### Preparation of Cu_7_S_4_ Nanocrystals

The Cu_7_S_4_ nanocrystals were prepared by a typical thermolysis method (Li et al., 2014). Firstly, CuCl_2_⋅2H_2_O (20 mmol) was dissolved into 10 mL deionized water, which was then dropwise added into aqueous solution of SDEDTC (90 mL, 50 mmol) under magnetically stirring. After stirring for 2 h, the above solution was centrifuged (5000 rpm, 5 min) and washed with deionized water. The precipitate [Cu(DEDTC)_2_ precursor] was dried in vacuum at 50°C for further use. Secondly, oleic acid (15 mL) and oleylamine (10 mL) were added into three-neck bottle and heated to 280°C within 30 min under the continuous nitrogen flow to remove any moisture and oxygen. The Cu(DEDTC)_2_ precursor (1 mmol) dissolved in 2 mL oleic acid was injected to the three-neck bottle and heated at 280°C for 10 min. The dark green mixture was quickly cooled to 60°C by air flow and then 20 mL ethanol was introduced to precipitate Cu_7_S_4_ nanocrystals. Finally, the Cu_7_S_4_ nanocrystals were dispersed in ethanol followed by centrifuging and washed with ethanol for three times.

### Preparation of Cu_7_S_4_@SiO_2_ Nanoplatforms

The Cu_7_S_4_@SiO_2_ nanoplatforms were prepared by *in situ* growing SiO_2_ shell on the surface of Cu_7_S_4_ nanocrystals. Firstly, the hydrophobic Cu_7_S_4_ nanocrystals were converted into hydrophilic Cu_7_S_4_ through the surface-modification with CTAB. The Cu_7_S_4_ nanocrystals in chloroform solution (5 mL, 10 mg/mL) were added into aqueous solution of CTAB (20 mL, 100 mg/mL), which was stirred vigorously at 40°C for 24 h and then centrifuged to collect hydrophilic Cu_7_S_4_ nanocrystals. Secondly, the hydrophilic Cu_7_S_4_ nanocrystals were dispersed into 50 mL deionized water, followed by the introduction of NaOH solution (0.1 mL, 10 mg/mL) and TEOS (0.1 mL) under sonication. After 1 h of sonication, then 100 μL of PEG-silane was added and the mixture was maintained at 40°C for another 8 h. The products were centrifuged and washed with deionized water for three times for collecting Cu_7_S_4_@SiO_2_ nanoplatforms.

### Characterizations

The size, morphology, phase of Cu_7_S_4_@SiO_2_ nanoplatforms were characterized by using JEOL 2100F transmission electron microscopy (TEM). The photoabsorption of Cu_7_S_4_@SiO_2_ nanoplatforms was studied on a Shimadzu UV-1900 spectrophotometer. The concentration of copper irons released from the Cu_7_S_4_@SiO_2_ nanoplatforms was determined by an inductively coupled plasma atomic emission spectroscopy (ICP-AES). The specific surface area and pore diameter of Cu_7_S_4_@SiO_2_ powder were investigated on the Autosorb-iQ/Autosorb-iQ Brunauer-Emmett-Teller (BET).

### Photothermal Conversion Efficiency

The photothermal performance of Cu_7_S_4_@SiO_2_ nanoplatforms was investigated by illuminating their aqueous dispersion at a series of concentrations under a 1064 nm laser with the output power density of 0.6 Wcm^–2^. The temperature change was recorded by using a thermal imaging camera. The photothermal conversion efficiency of Cu_7_S_4_@SiO_2_ was calculated according to the previous report (Li et al., 2014) by applying the below equations:

(1) $$\eta =\frac{hA(\Delta T_{\max,\text{dis}}-\Delta {\text{T}}_{\max,{\text{H}}_{2}\,\text{O}}}{I\,\left(1-10^{-A_{1064}}\right)}$$

(2) $$\tau_{s}=\frac{m_{D}\,C_{D}}{h\,A}$$

Where *I* and *A*_106__4_, respectively, stand for the NIR laser intensity and the absorbance at 1064 nm. Δ*T*_max,dis_ and Δ*T*_max,H_2O_ are the temperature change of deionized water and the solution containing Cu_7_S_4_@SiO_2_ nanoplatforms. The *h* and *A* are the heat transfer coefficient and the surface area, and the value of *hA* is determined from Eq. 2 by using the system time constant *τ_*s*_* with the help of the mass (*m*_D_) and the heat capacity (*C*_*D*_) of deionized water.

### Dox Loading and Releasing

For loading DOX, Cu_7_S_4_@SiO_2_ (2.5 mL, 10 mg/mL) and DOX (10 mg) were dispersed into PBS solution, which was magnetically stirred in the dark. After 24 h of stir, the mixture was centrifuged (12,000 rpm, 30 min) and the supernatant was collected. The DOX loading content was calculated based on (load weight of DOX/Cu_7_S_4_@SiO_2_/DOX) × 100%, in which the load weight of DOX was determined by (10 mg – DOX in the supernatant). For DOX releasing, Cu_7_S_4_@SiO_2_/DOX was dispersed into PBS at pH 7.4 or pH 5.4 and divided into two groups. One group was used as control and 0.5 mL solution were taken out which was centrifuged (12,000 rpm, 15 min) at each time point. Another group was irradiated by a 1064 nm NIR laser (0.6 Wcm^–2^) at a specific time. The DOX releasing rate was calculated by using (the release weight of DOX/the load weight of DOX) × 100%.

### Photothermal-Chemo Therapy *in vitro*

Melanoma cells were seeded into a 96-well plate (1.2 × 10^4^ cells per well) at 37°C in the presence of 5% CO_2_ for 24 h. After incubation, the cell medium was removed, the cells were divided into four groups: (1) control, (2) DOX, (3) Cu_7_S_4_@SiO_2_/DOX+NIR, (4) Cu_7_S_4_@SiO_2_+NIR. Hundred microliter of the dispersion at varied concentrations was then added into the wells. After incubation for another 24 h, the cells were washed with PBS buffer solution for three times. Then the cells were irradiated with/without a 1064 nm laser. Cell viability was measured using the CCK-8 assay.

To visually compare the viability difference in cellular level among four groups, cells were seeded into a 24-well plate at a density of 1.2 × 10^5^ cells per well. After the cells in the four groups were treated, the cells were stained with calcein AM (live cells) and propidium iodide (dead cells) to distinguish live cells with green fluorescence and dead cells with red fluorescence.

### Photothermal-Chemo Therapy *in vivo* and Histological Examination

The BALB/c mice (∼16 g, male) with 4T1 tumors (the surface diameter of 0.3∼0.5 cm) on the back were divided into four groups (*n* = 3): (1) The control group; (2) DOX group; (3) Cu_7_S_4_@SiO_2_+NIR group; (4) Cu_7_S_4_@SiO_2_/DOX+NIR group. The mice in (2) group were intratumorally injected with DOX PBS solution (50 μL, 80 μg), and mice in (3 and 4) group were, respectively, injected with Cu_7_S_4_@SiO_2_ (50 μL, 0.1 mg mL^–1^) or Cu_7_S_4_@SiO_2_/DOX PBS solution (50 μL, 0.1 mg mL^–1^). The tumors on mice in (3, 4) group were exposed to a 1064 nm NIR laser (0.6 Wcm^–2^) at the 0.5 h post-injection and mice body. After treatments, mice in all groups were observed regarding their body weight and tumor sizes. When a tumor size was beyond 1.0 cm, mice in all groups were sacrificed and tumors were extracted for histological examination.

## Results and Discussion

**FIGURE 1:**
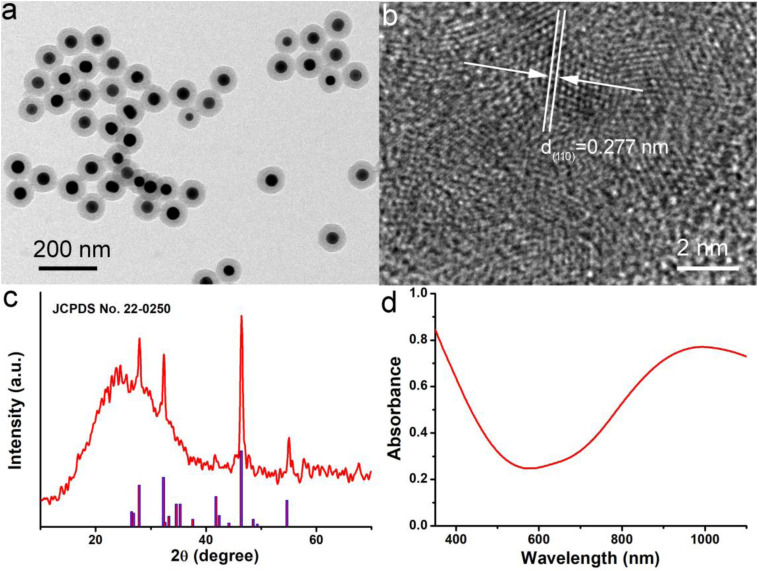
**(a)** TEM image of Cu_7_S_4_@SiO_2_ nanoplatforms showing the Cu_7_S_4_ core and mesoporous SiO_2_ shell. **(b)** HR-TEM image of Cu_7_S_4_ core with the clear lattice. **(c)** XRD pattern of Cu_7_S_4_@SiO_2_ nanoparticles. **(d)** The typical UV-vis-NIR photoabsorption spectrum of Cu_7_S_4_@SiO_2_ dispersed in deionized water.

The Cu_7_S_4_@SiO_2_ nanoplatforms were prepared by *in situ* growing SiO_2_ shell on the surface of Cu_7_S_4_ nanocrystals. Firstly, Cu_7_S_4_ nanocrystals were synthesized through a typical thermolysis method, where the Cu(DEDTC)_2_ precursor with Cu source and S source was heated to 280°C for 10 min to produce uniform Cu_7_S_4_ nanocrystals. Secondly, the Cu_7_S_4_ nanocrystals were served as the core to allow the coating of SiO_2_ shell through the hydrolysis of TEOS. During the SiO_2_ coating, CTAB were used as a soft template. SiO_2_ grew around the template due to electrostatic interactions. Supplementary Figure S1 shows the size and zeta potential of nanoparticles during the process of synthesis. FTIR spectra in Supplementary Figure S2 demonstrated that PEG was coated on the surface of Cu7S4@SiO_2_ nanoplatforms, thus the nanoplatforms could be realized the bioapplication directly. The as-obtained Cu_7_S_4_@SiO_2_ nanoplatforms have a uniform morphology and the average size of 100 nm, as shown in the TEM image (Figure 1a). Obviously, the Cu_7_S_4_@SiO_2_ nanoplatforms consist of Cu_7_S_4_ as the core and SiO_2_ as the shell, and the Cu_7_S_4_ nanocrystals have the average diameter of 50 nm and the SiO_2_ shells have the average thickness of 25 nm. The nanoplatforms showed good dispersion as the size in water showed little change over time demonstrated by dynamic light scattering (DLS, Supplementary Figure S3). Furthermore, the high-resolution (HR-TEM) image in Figure 1b demonstrates the apparent lattice with an interplane *d* spacing of ∼0.277 nm, which can be indexed to the (110) plane of the orthorhombic Cu_7_S_4_ (JCPDS card no. 22-0250), which verifies the core is Cu_7_S_4_ nanocrystals. Subsequently, the phase of Cu_7_S_4_@SiO_2_ powders was characterized by using XRD. The XRD pattern (Figure 1c) reveals that there are four prominent peaks centered at 27.87°, 32.30°, 46.40°, and 54.70°, which can be respectively, corresponded to the (202), (220), (224), and (422) planes for the orthorhombic Cu_7_S_4_ (JCPDS card no. 22-0250). It should be noted that there is a broad peak between 10° and 40°, which should be attributed to the characteristic peak originating from the amorphous SiO_2_ shell. Supplementary Figure S4 shows the XPS spectra of Cu 2p in the Cu_7_S_4_@SiO_2_ nanoparticles. The binding energy peaks at 932.8 and 954.6 eV can be assigned to Cu^+^ coordinated to Cu in Cu_7_S_4_@SiO_2_ nanoparticles, whereas the binding energy peak at 943.2 eV is formally described as Cu^2+^. The coexistence of Cu^+^ and Cu^2+^ indicated the Cu vacancies on the surface of Cu_7_S_4_ nanocrystals (Li et al., 2015). Thus, the above results confirmed the successful preparation of Cu_7_S_4_@SiO_2_ nanoplatforms with Cu_7_S_4_ core and SiO_2_ shell.

Subsequently, the photoabsorption of Cu_7_S_4_@SiO_2_ nanoplatforms was studied by using UV-vis-NIR spectrometer. The Cu_7_S_4_@SiO_2_ nanoplatforms can be well dispersed into deionized water and their aqueous solution shows the dark green. As demonstrated in UV-vis-NIR photoabsorption spectra (Figure 1d), the aqueous solution containing Cu_7_S_4_@SiO_2_ nanoplatforms exhibits the strong absorption in the UV-vis region with the edge at ∼590 nm, which can be ascribed to the bandgap absorption of Cu_7_S_4_ as a typical semiconductor. Importantly, the aqueous solution of Cu_7_S_4_@SiO_2_ demonstrates a strong and broad NIR absorption (>650 nm) and the absorption intensity goes up with the increase of wavelength up to 1100 nm. Compared to the bandgap-induced UV-vis absorption, this kind of NIR absorption should be attributed to the localized surface plasmon resonances (LSPR) effect because of Cu vacancies on the surface of Cu_7_S_4_ nanocrystals, which has been reported for other Cu_2–x_S nanocrystals (Zhao et al., 2009; Luther et al., 2011). By determining the Cu_7_S_4_@SiO_2_ nanoparticle concentration via ICP-AES, the extinction coefficient of the nanoparticles at 1064 nm was measured to be 13.9 L g^–1^ cm^–1^, which was higher than the that of cobalt/manganese oxide (CMO) nanocrystals (Liu et al., 2019). Thus, Cu_7_S_4_@SiO_2_ nanoplatforms are capable of strong and broad NIR photoabsorption because of Cu_7_S_4_ core.

**FIGURE 2:**
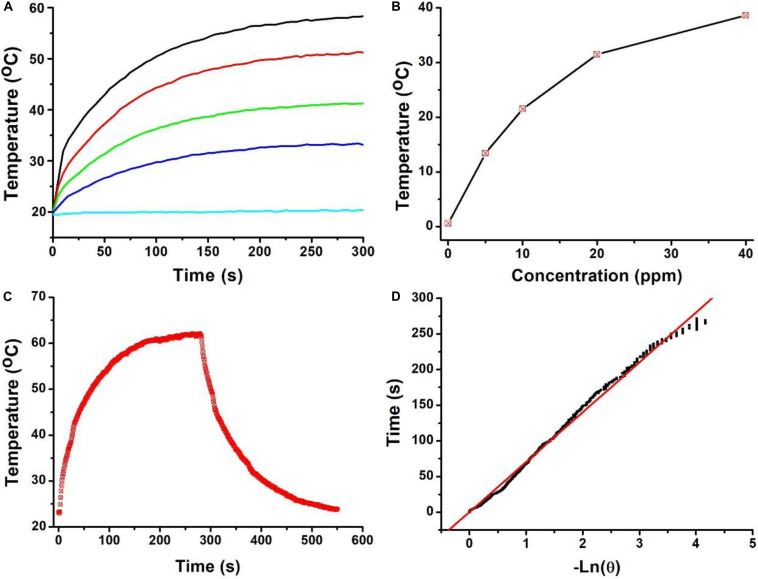
**(A)** Temperature curves of solution containing the Cu_7_S_4_@SiO_2_ nanoparticles with the Cu concentration of 0–40 ppm. **(B)** The Temperature elevation versus the concentrations of Cu_7_S_4_@SiO_2_ nanoparticles. **(C)** Temperature curve of a solution containing the Cu_7_S_4_@SiO_2_ nanoparticles with 1064 nm NIR laser on/off. **(D)** The linear fit of time data with ln(θ) to obtain system time constant *τ_*s*_*.

Owing to the strong NIR absorption, we further explored the photothermal performance of Cu_7_S_4_@SiO_2_ nanoparticles. The wavelength of NIR laser is quite important for photothermal therapy, and two biological transparency windows are reported as NIR-I (650–950 nm) and NIR-II (1000–1350 nm). Compared to the commonly used 808 nm, 915 m and 980 nm NIR laser, 1064 nm NIR laser can offer more efficient tissue penetration depth by considering absorption and scattering effects (Tsai et al., 2013). Therefore, we selected 1064 nm NIR laser to study the photothermal effect of Cu_7_S_4_@SiO_2_ nanoparticles. When exposed to a 1064 nm NIR laser at the intensity of 0.6 W cm^–2^, the temperature of deionized water increases slightly (∼1.3°C) within 5 min of irradiation, which confirmed the negligible photothermal effect from deionized water (Figure 2A). In contrast, once exposed to laser, the aqueous solutions containing Cu_7_S_4_@SiO_2_ nanoparticles exhibit rapid temperature elevation within 120 s and then show a slow temperature elevation due to the balance between heat production and loss. Figure 2B summarizes the temperature elevation versus to the concentration, and they are determined to be 14.2, 21.8, 31.5, and 39.4°C for the concentration of 10, 20, 30, and 40 ppm, respectively. To further clarify the photothermal performance of Cu_7_S_4_@SiO_2_ nanoparticles, we carried out an experiment to calculate its photothermal conversion efficiency. The aqueous solution of Cu_7_S_4_@SiO_2_ nanoparticles were subjected to a 1064 nm NIR laser on/off, and the whole temperature change was recorded in Figure 2C. The system time constant *τ_*s*_* can be obtained by linearly plotting the time data with ln(θ), as shown in Figure 2D. By referencing to the previous reports, the photothermal conversion efficiency of Cu_7_S_4_@SiO_2_ nanoparticles is 48.2% which is comparable to the reported CuS nanomaterials (Li et al., 2014). Therefore, the Cu_7_S_4_@SiO_2_ nanoparticles can rapidly and efficiently convert 1064 nm NIR laser energy into heat with the concentration-relied photothermal performance.

**FIGURE 3:**
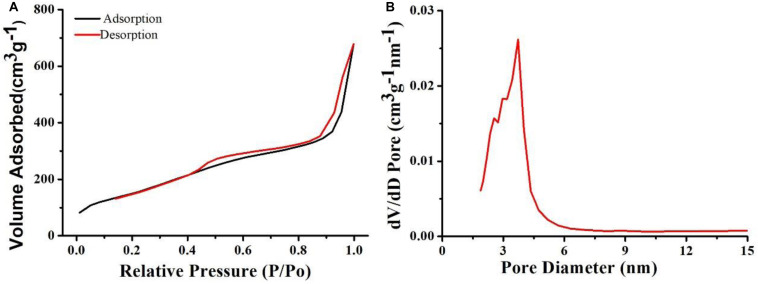
**(A)** The nitrogen adsorption-desorption curve of Cu_7_S_4_@SiO_2_ nanoparticles. **(B)** The pore diameter of Cu_7_S_4_@SiO_2_ nanoparticles.

After demonstrating the photothermal performance of Cu_7_S_4_@SiO_2_ nanoparticles, we evaluated their DOX loading capacity due to the SiO_2_ shell. Prior to loading DOX, the Brunauer-Emmett-Teller (BET) surface area and pore size were investigated by using nitrogen adsorption-desorption curves. The nitrogen adsorption/desorption isotherms (Figure 3A) illustrate that the Cu_7_S_4_@SiO_2_ nanoparticles have a specific surface area of 125.9 m^2^/g, showing the high surface area. In addition, the pore diameter of Cu_7_S_4_@SiO_2_ nanoparticles was also recorded and the average pore diameter was determined to be ∼4.5 nm (Figure 3B). Therefore, the mesoporous SiO_2_ shell confers high specific surface area and pores, which will facilitate the following DOX loading.

**FIGURE 4:**
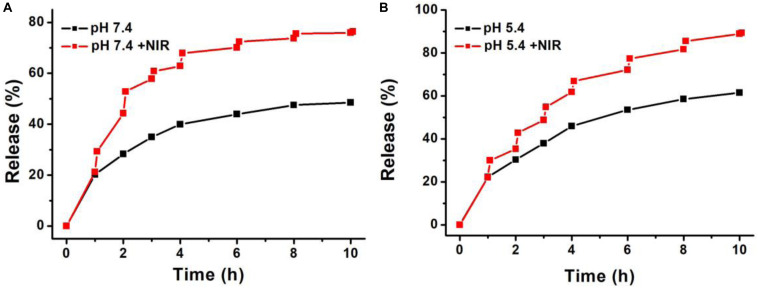
The DOX-releasing profile of Cu_7_S_4_@SiO_2_/DOX in **(A)** PBS solution (pH = 7.4) and **(B)** acetate buffer (pH 5.4) in the absence or presence of 1064 nm NIR laser.

The release ability of DOX from Cu_7_S_4_@SiO_2_/DOX (the DOX loading content is 29.8%) was studied by dispersing Cu_7_S_4_@SiO_2_/DOX into PBS solution. At the time point of 1, 2, 3, 4, 6, 8, and 10 h, part of solution was taken out from the original 10 mL dispersion and centrifuged for collecting the released DOX in the supernatant, and the amount of DOX in the supernatant was calculated by applying absorption-concentration curve. Figure 4A shows the DOX releasing profile at pH 7.4, and it is clear that, with the prolong of time, the DOX releasing rate unceasingly goes up which can be determined to be 19.5% at 1 h, 28.4% at 2 h, and 39.4% at 4 h. After 4 h, the DOX releasing rate becomes very slow. In order to study the effect of NIR laser-induced photothermal effect on the DOX releasing rate, we irradiated the Cu_7_S_4_@SiO_2_/DOX dispersion at the time point of 1, 2, 3, 4, 6, 8, and 10 h by using a 1064 nm NIR laser (5 min, 0.6 W cm^–2^). For instance, after the firstly irradiation, the DOX releasing rate is calculated to be 45.8% which is much higher than that (28.4%) at the 2 h, indicating NIR laser-induced photothermal effect can significantly enhance the DOX releasing rate. After the irradiation cycles, 75.6% of DOX is released from Cu_7_S_4_@SiO_2_/DOX with the help of 1064 nm NIR laser in comparison to 47.4% of the released DOX without irradiation, within 10 h. Thereby, it is concluded that the Cu_7_S_4_ nanocrystals within Cu_7_S_4_@SiO_2_/DOX can produce heat to accelerate the DOX release. To study the DOX release behavior from Cu_7_S_4_@SiO_2_/DOX at the simulating tumor microenvironment, the release of the DOX against buffer solution at pH 5.4 was measured. It was found that the drug release rate became much faster at pH 5.4 (Figure 4B) due to the increase in the solubility of the DOX under acidic conditions, which is beneficial for cancer therapy since the microenvironments of extracellular tissues of tumors and intracellular lysosomes and endosomes are acidic.

**FIGURE 5:**
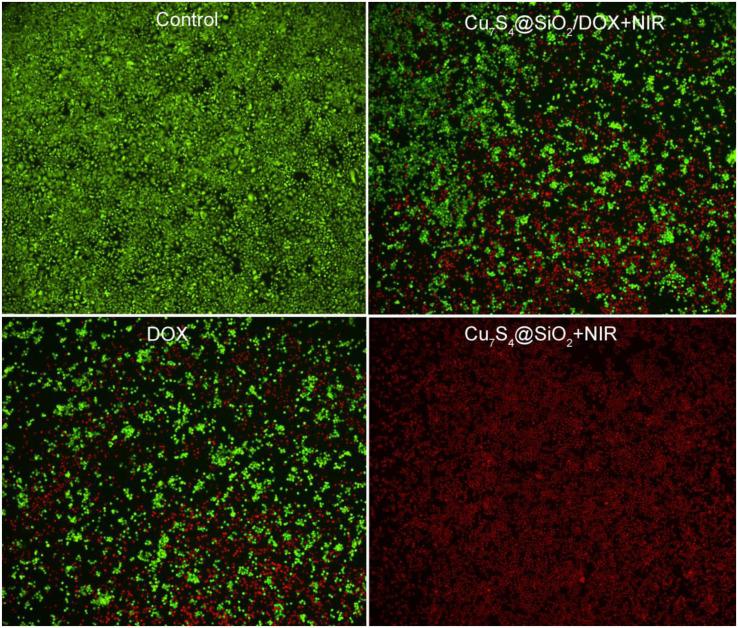
Confocal images of calcein AM (green, live cells) and propidium iodide (red, dead cells) co-stained cells after incubation with Cu_7_S_4_@SiO_2_/DOX for different treatments. Magnification: 100 times.

Giving the efficient photothermal conversion effect, the high DOX loading capacity and the NIR-enhanced DOX releasing, Cu_7_S_4_@SiO_2_/DOX can be used as excellent nanoagents for photothermal-chemo therapy. To demonstrate the synergy effect of photothermal-chemo therapy, the combination index of Cu_7_S_4_@SiO_2_/DOX nanoparticles was measured according to previous work (Wang et al., 2013). The half-maximal inhibitory concentration (IC50) of cancer cells incubated with Cu_7_S_4_@SiO_2_/DOX nanoparticles for combination photothermal-chemo therapy is 0.52 mg mL^–1^, while the IC50 for the sole photothermal therapy and chemotherapy is 0.67 and 1.35 mg mL^–1^, respectively. The combination index was calculated to evaluate the combination effect of different therapies and found to be 0.823, which demonstrated the synergistic effect of Cu_7_S_4_@SiO_2_/DOX nanoparticles for photothermal-chemo therapy. Additionally, to visualize the efficiency of photothermal-chemo therapy, cells after the indicated treatments were co-stained with calcein-AM and propidium iodide (PI, Figure 5). The results further demonstrated the synergetic effect.

**FIGURE 6:**
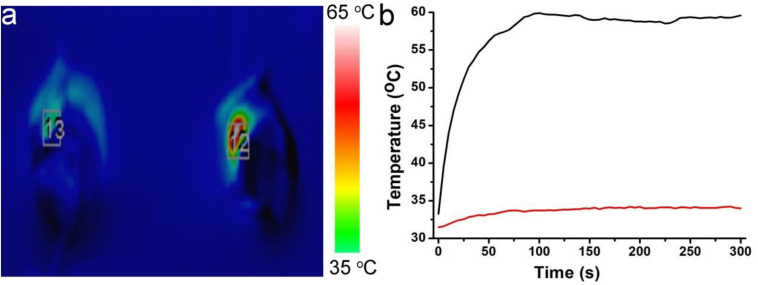
**(a)** The thermal image of mice after intratumorally injecting with Cu_7_S_4_@SiO_2_/DOX PBS solution (right) or PBS solution (left) under the irradiation of 1064 nm NIR laser at the power density of 0.6 Wcm^– 2^. **(b)** The temperature curves showing the temperature change in tumor area.

Before realizing bioapplication, the Cu^2+^ release of Cu_7_S_4_@SiO_2_ nanoparticles in PBS was evaluated because of Cu^2+^ being toxic. It was revealed that the concentration of the released Cu^2+^ from Cu_7_S_4_@SiO_2_ nanoparticles was very low (Supplementary Figure S5), which caused almost no toxicity. We then evaluated the toxicity *in vivo* of Cu_7_S_4_@SiO_2_. In the experimental group, the material was injected intravenously into mice, while mice in the control group were injected with PBS. After a month, HE analysis of the main organs of mice in the two groups showed no significant difference (Supplementary Figure S6). To perform photothermal-chemo therapy of tumors, mice bearing with melanoma tumor were randomly allocated into four groups as follow: (1) The control group; (2) DOX group; (3) Cu_7_S_4_@SiO_2_+NIR group; (4) Cu_7_S_4_@SiO_2_/DOX+NIR group. The mice in (2) group were intratumorally injected with DOX PBS solution (50 μL, 80 μg), and mice in (3 and 4) group were intratumorally injected with Cu_7_S_4_@SiO_2_ (50 μL, 0.1 mg mL^–1^) and Cu_7_S_4_@SiO_2_/DOX PBS solution (50 μL, 0.1 mg mL^–1^). Figure 6a shows the typical thermal image of mice with the tumor area exposed to a 1064 nm NIR laser (0.6 Wcm^–2^), in which the tumor treated with Cu_7_S_4_@SiO_2_/DOX shows the bright red while the tumor received PBS exhibits the normal color. The surface temperature of tumor treated with Cu_7_S_4_@SiO_2_/DOX increases from ∼34.2°C to the balanced ∼57.9 °C at 300 s, resulting in temperature elevation of 23.7°C which was much higher than that (3.5°C) for the tumor received PBS (Figure 6b). Thus, the Cu_7_S_4_@SiO_2_/DOX within tumor remain high photothermal conversion effect, which can convert 1064 nm NIR laser energy into enough heat to thermally ablate cancer cells.

**FIGURE 7:**
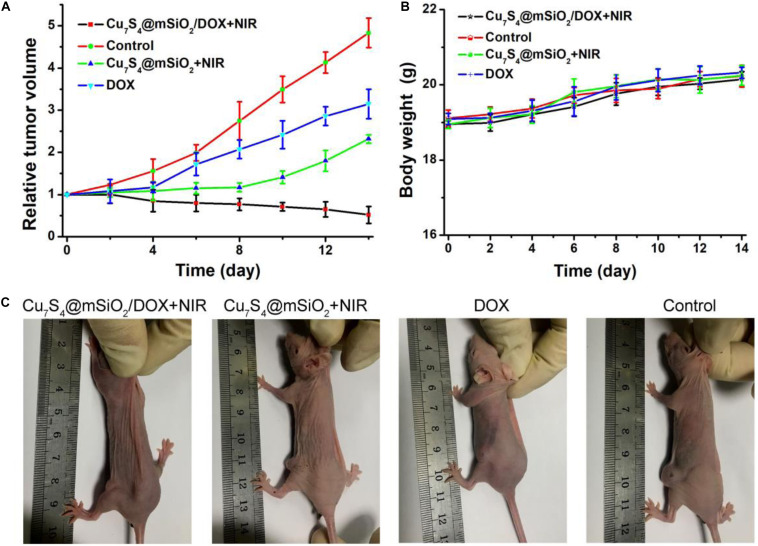
**(A)** The relative tumor volumes of mice after different treatments over a period of 14 days. **(B)** The body weight of mice in these groups. **(C)** The typical photographs of mice at the 14th day.

After different treatments, mice in all groups were raised under the standard condition for the long-term observation of cancer treatment efficacy. The tumor sizes and body weights were recorded. The change of relative tumor volumes is demonstrated in Figure 7A. Obviously, the tumor volume in the control group increases greatly which was five times the initial volume. For the tumors in the DOX group and Cu_7_S_4_@SiO_2_+NIR group, their growth has been inhibited, due to the cytotoxicity of DOX for DOX group and the photothermal therapy for Cu_7_S_4_@SiO_2_+NIR group. Interestingly, in the case of the tumors in the Cu_7_S_4_@SiO_2_/DOX+NIR group, their volume goes down continuously with the significant inhibition efficiency compared to the other three groups. The high inhibition efficiency should be due to the synergistic photothermal-chemo effect from the combination of Cu_7_S_4_@SiO_2_/DOX and NIR laser irradiation. On the one side, the photothermal therapy can instantly kill part of cancer cells through high temperature, and on the other side, the load DOX within Cu_7_S_4_@SiO_2_/DOX can continuous release to kill the remaining cancer cells, thus achieving the synergistic effect on melanoma tumors. In addition, there was no any sign of loss in body weight for all groups (Figure 7B). Therefore, the satisfactory inhibition efficiency can be realized from the synergistic photothermal-chemo therapy by the combination of Cu_7_S_4_@SiO_2_/DOX and 1064 nm NIR laser, while no obvious side effect for mice.

**FIGURE 8:**
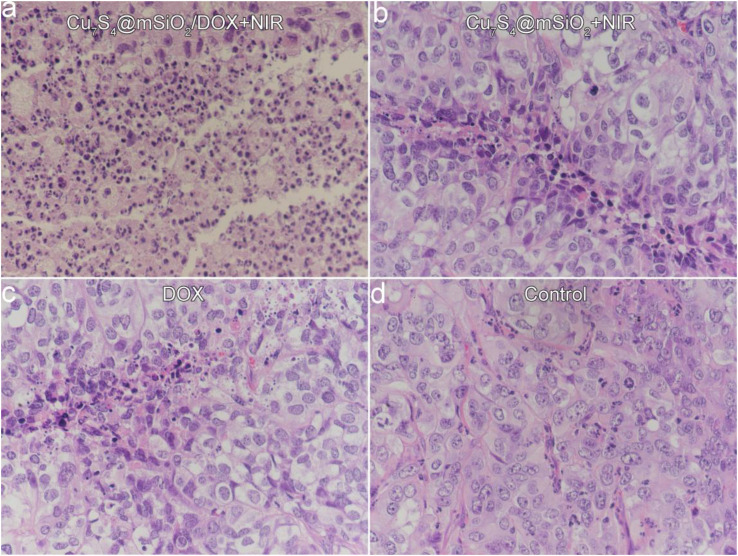
The typical images of tumor slices stained with H&E assay for **(a)** Cu_7_S_4_@SiO_2_/DOX+NIR group, **(b)** Cu_7_S_4_@SiO_2_+NIR group, **(c)** DOX group, and **(d)** Control group.

Furthermore, mice in all groups were sacrificed when a tumor size was beyond 1.0 cm (Figure 7C). The tumors were extracted for the sacrificed mice, which were embedded in paraffin and crysectioned into slices. After stained with H&E assay, the tumor slices were imaged for histological examination. Figure 8 manifests the typical morphology of tumor cells, and the cancer cells shows the normal and complete morphology in regard to the cell size, shape and nuclear. The significant cell damage is noticed for Cu_7_S_4_@SiO_2_/DOX+NIR group (Figure 8a), including the destroyed cell membranes and the condensed nucleus. For the tumor cells in the Cu7S4@SiO_2_+NIR group (Figure 8b) and DOX group (Figure 8c), there are some cells showing the destroyed morphology, indicating the limited therapeutical effect through photothermal therapy or chemotherapy. On the contrary, the cell size and shape showed no difference in the control group (Figure 8d). The above histological examination solidly verifies the higher therapeutical effect of photothermal-chemo therapy than photothermal therapy or chemotherapy.

## Conclusion

In summary, we prepared the Cu_7_S_4_@SiO_2_/DOX and used them as efficient nanoplatforms for synergistic photothermal-chemo therapy on melanoma tumors. The Cu_7_S_4_@SiO_2_/DOX was prepared by firstly synthesizing Cu_7_S_4_ nanocrystals, then *in situ* growing SiO_2_ shell on the surface of Cu_7_S_4_ nanocrystals, and finally loading DOX. The Cu_7_S_4_@SiO_2_ consisted of Cu_7_S_4_ core with the average diameter of 50 nm and SiO_2_ shell with the average thickness of 25 nm. The Cu_7_S_4_@SiO_2_ nanoplatforms exhibited the strong and broad NIR absorption and rapidly converted 1064 nm laser energy into heat, and they also demonstrated high specific surface area and a large amount of pores with high DOX-loading content of 59.8%. Importantly, under the irradiation of 1064 nm laser, Cu_7_S_4_@SiO_2_/DOX simultaneous generated heat and accelerated the DOX release. Give these advantages, mice were intratumorally injected with Cu_7_S_4_@SiO_2_/DOX and irradiated with1064 nm laser, which achieved the highest therapeutical effect through synergistic photothermal-chemo therapy compared to photothermal therapy or chemotherapy alone. Therefore, the Cu_7_S_4_@SiO_2_/DOX can be served as novel and efficient photothermal-chemo nanoagents for efficient tumor therapy.

## Data Availability Statement

All datasets presented in this study are included in the article/Supplementary Material.

## Ethics Statement

The animal study was reviewed and approved by the Ninth People’s Hospital, Shanghai Jiao Tong University School of Medicine.

## Author Contributions

LZ and HP contributed equally to this work. LZ, HP, and XH designed the project and wrote the manuscript. LZ, HP, and YL carried out the experiment. LZ and FL performed the experimental data analysis. All the authors contributed to discussion of the results.

## Conflict of Interest

The authors declare that the research was conducted in the absence of any commercial or financial relationships that could be construed as a potential conflict of interest. The reviewer XH declared a shared affiliation, with no collaboration, with the authors to the handling editor.
