# Supplementary material for: Constructing Cu7S4@SiO2/DOX Multifunctional Nanoplatforms for Synergistic Photothermal–Chemotherapy on Melanoma Tumors
Source: Front Bioeng Biotechnol. 2020 Sep 15;8:579439. doi: 10.3389/fbioe.2020.579439 (PMC7522791; doi:10.3389/fbioe.2020.579439)
Supplement: Supplementary file 1 [file Image_1.pdf]

Supporting Information

Constructing $Cu_7S_4@SiO_2$/DOX multifunctional nanoplatforms for synergistic photothermal-chemo therapy on melanoma tumors

Leilei Zhang,[1,2] Hui Pan,[1,2] Yongyun Li,[1,2] Fang Li,[1,2] Xiaolin Huang[1,2]*

[1] Department of Ophthalmology, Ninth People's Hospital, Shanghai JiaoTong University School of Medicine, Shanghai 200011, China

[2] Shanghai Key Laboratory of Orbital Diseases and Ocular Oncology, Shanghai 200011, China.

*Correspondence:

Xiaolin Huang, Email: drmaureenhuang@163.com.

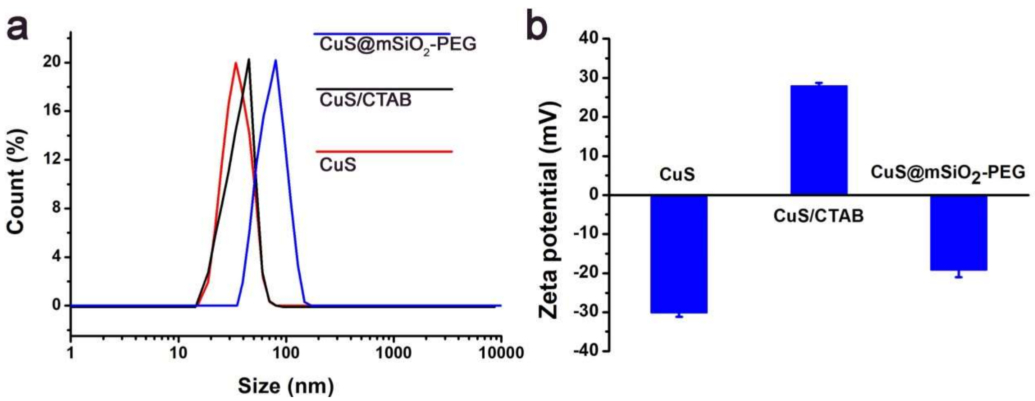

Figure S1. **The size (a) and zeta potential (b) of CuS, CuS/CTAB and $CuS@mSiO_2$-PEG nanoparticles.**

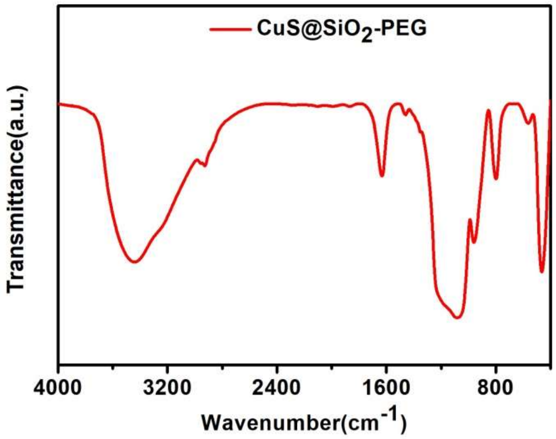

Figure S2. FTIR spectra of CuS@SiO_2-PEG.

The sample shows a broad band at 3432 cm^{-1} due to OH stretching; the band at 2931 cm^{-1} are corresponding to CH_2 symmetric stretching, respectively; the band at 1635 cm^{-1} are assigned to C=O symmetric stretching; the band at 1471 cm^{-1} corresponds to CH_2 scissoring vibrations; In addition, the band at 1082 cm^{-1} is contributed to C-O stretching vibration coordinating to metal cations. These results indicated that the surface polymer coated on CuS@SiO_2 was PEG.

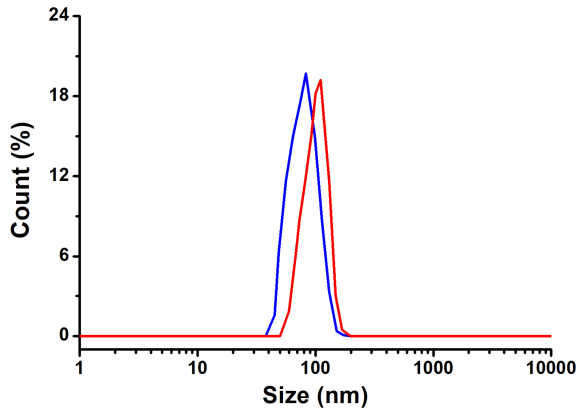

Figure S3. Dynamic light scattering (DLS) data of as-prepared (blue line) and stored (in water for 7 days) $Cu_7S_4@SiO_2$ core-shell nanoparticles.

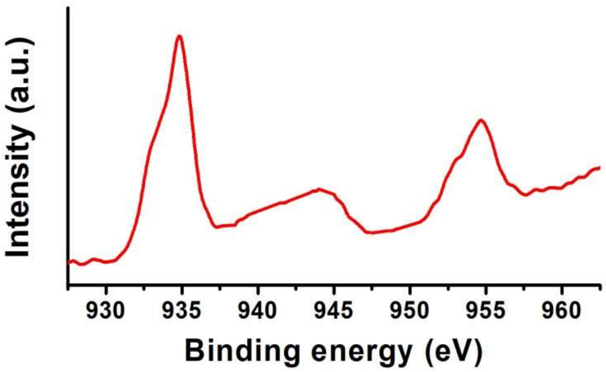

Figure S4. XPS spectra of Cu 2p in the $Cu_7S_4@SiO_2$ nanoparticles.

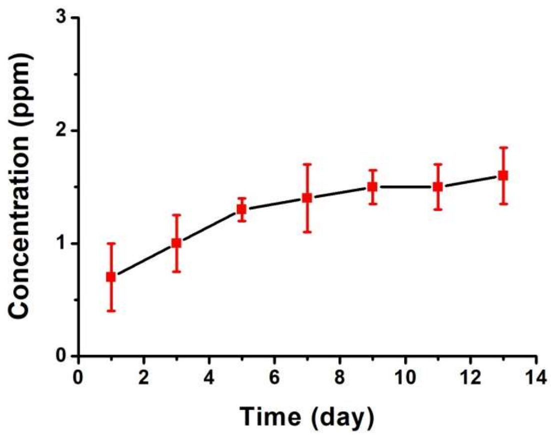

Figure S5. The Cu^{2+} release of $Cu_7S_4@SiO_2$ nanoparticles (2 mg/mL) in PBS.

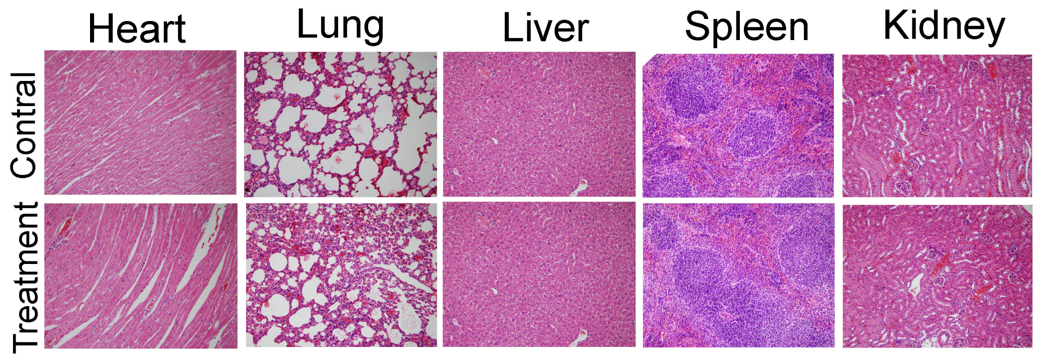

Figure S6. H&E stained slices of main organs. Magnification: 200 times.
